# Downregulation of M-channels in lateral habenula mediates hyperalgesia during alcohol withdrawal in rats

**DOI:** 10.1038/s41598-018-38393-7

**Published:** 2019-02-25

**Authors:** Seungwoo Kang, Jing Li, Wanhong Zuo, Pei Chen, Danielle Gregor, Rao Fu, Xiao Han, Alex Bekker, Jiang-Hong Ye

**Affiliations:** 1 Department of Anesthesiology, Rutgers, The State University of New Jersey, New Jersey Medical School, Newark, New Jersey USA; 2 Pharmacology, Physiology, and Neuroscience, Rutgers, The State University of New Jersey, New Jersey Medical School, Newark, New Jersey USA; 3 Department of Molecular Pharmacology and Experimental Therapeutics, Mayo Clinic College of Medicine, Rochester, Minnesota USA

## Abstract

Hyperalgesia often occurs in alcoholics, especially during abstinence, yet the underlying mechanisms remain elusive. The lateral habenula (LHb) has been implicated in the pathophysiology of pain and alcohol use disorders. Suppression of m-type potassium channels (M-channels) has been found to contribute to the hyperactivity of LHb neurons of rats withdrawn from chronic alcohol administration. Here, we provided evidence that LHb M-channels may contribute to hyperalgesia. Compared to alcohol naïve counterparts, in male Long-Evans rats at 24-hours withdrawal from alcohol administration under the intermittent access paradigm for eight weeks, hyperalgesia was evident (as measured by paw withdrawal latencies in the Hargreaves Test), which was accompanied with higher basal activities of LHb neurons in brain slices, and lower M-channel protein expression. Inhibition of LHb neurons by chemogenetics, or pharmacological activation of M-channels, as well as overexpression of M-channels’ subunit KCNQ3, relieved hyperalgesia and decreased relapse-like alcohol consumption. In contrast, chemogenetic activation of LHb neurons induced hyperalgesia in alcohol-naive rats. These data reveal a central role for the LHb in hyperalgesia during alcohol withdrawal, which may be due in part to the suppression of M-channels and, thus, highlights M-channels in the LHb as a potential therapeutic target for hyperalgesia in alcoholics.

## Introduction

A major challenge faced by the alcohol researchers is to find better strategies for the treatment/prevention of relapse drinking. Hyperalgesia, an increased sensitivity to pain, which has frequently occurred in alcoholics and is exacerbated during withdrawal, is an important contributor to relapse^1–4^. This has been observed in human alcoholics^5^ and animal models of chronic alcohol consumption^6–9^. Conversely, chronic pain states could affect alcohol use patterns, contributing to the acquisition and maintenance of problematic drinking^10,11^.

The fact that neuronal substrates and pathways for pain transmission overlap with those for negative reinforcement in alcohol addiction may underlie the observation that chronic pain states significantly affect alcohol use patterns leading to dependence and *vice versa*^12,13^. Therefore, adaptation of neuronal pathways due to long-term alcohol intoxication and dependence may alter pain perception and exacerbate underlying chronic pain. However, the fundamental molecular and cellular mechanisms contributing to hyperalgesia occurring during ethanol withdrawal remains unclear.

The lateral habenula (LHb), a small epithalamic brain area, transfers information from the limbic forebrain to monoaminergic centers such as the serotoninergic raphe, the dopaminergic ventral tegmental area (VTA), and the norepinephrinergic locus coeruleus^14,15^. The LHb has been spotlighted because of its integrated role in pathophysiological behaviors involving stress, pain, sleep, cognition, reward, and emotion^16–18^. LHb disturbances have been implicated in the pathogenic interrelation between pain and psychiatric disorders^18–20^. Indeed, pharmacological inhibition of activity of LHb neurons is analgesic^9,21^. A study indicates that reduction of LHb M-channels, a voltage-gated potassium channel contributes to increased excitability of LHb neurons of ethanol-withdrawn rats^22^. However, it remains unclear whether hyperalgesia in ethanol-withdrawn rats involves adaptations in the LHb, as well as the M-channels.

In the current study, we addressed these questions in rats by using a combination of chemogenetic, electrophysiological, pharmacological, genetic, and behavioral approaches. The results showed that activation of M-channels, as well as overexpression of M-channels’ subunit KCNQ3, reduced the pain sensitivity and relapse-like alcohol consumption. Thus, LHb M-channels could be proposed as a potential therapeutic target for hyperalgesia in alcoholics.

## Results

### Hyperalgesia in ethanol-withdrawn rats is accompanied with hyperactivity of LHb neurons in brain slices

**Figure 1:**
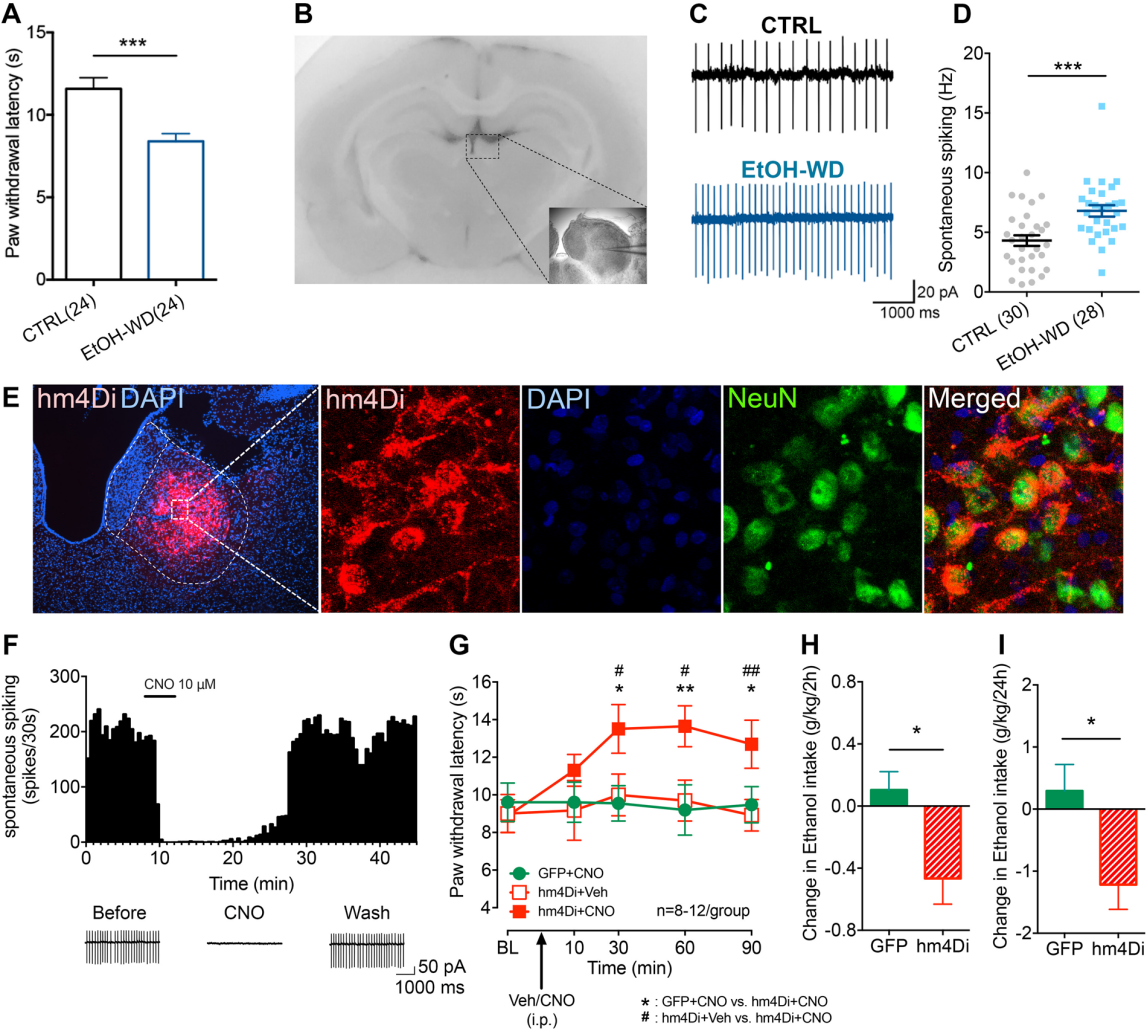
Chemogenetic inhibition of LHb neurons alleviates hyperalgesia of rats withdrawn from chronic repeated voluntary ethanol drinking. (**A**) Mean paw withdrawal latency is significantly shorter in rats at 24 h withdrawal from voluntary drinking ethanol in the intermittent two-bottle choice paradigm (EtOH-WD) in comparison to ethanol-naïve control rats (CTRL). Unpaired *t*-test, *t* = 3.9, ***p < 0.001. N_rat_ = 24/group. (**B**) The schematic of location of recorded neurons. A camera captured image of the coronal section containing the LHb (box) and CCD camera captured IR image of the LHb for lose cell-attached patch-clamping recording (right corner). (**C**,**D**) Representative traces (**C**) and summary data (**D**) show increased spontaneous firing of LHb neurons in ethanol-withdrawn rats. Unpaired *t*-test, *t* = 3.789, ***p < 0.001. Numbers of neurons are indicated. (**E**) hm4Di-mCherry expression in LHb neurons after viral vector injection and immunofluorescence of the neuronal marker NeuN. A strong hM4Di-mCherry expression overlaps with NeuN. (**F**) Bath-applied CNO (10 μM) sharply inhibited spontaneous firings of LHb neurons infected with AAV-CaMKIIa-hM4Di-mCherry viruses. (**G**) After systemic CNO injection (1 mg/kg, i.p.), EtOH-WD rats infected with hm4Di showed an increased paw withdrawal latency than those infected with eGFP. Two-way ANOVA, for group F_2,29_ = 4.021, P = 0.0288, *post-hoc*: *p < 0.05, **p < 0.01 vs GFP + CNO; ^#^p < 0.05, ^##^p < 0.01 vs. hm4Di + Veh; N_rat_ = 8–12/group. (**H**,**I**) Pretreating hM4Di-rats with systemic injection of CNO (1 mg/kg, i.p.) reduced ethanol intake relative to saline injection, whereas eGFP hM3Dq mice were unaffected. For 2 h, Unpaired *t*-test, *t* = 2.796, *p < 0.05, Fig. 2D, N_rat_ = 8/group; For 24 h, Unpaired *t*-test, *t* = 2.587, *p < 0.05, N_rat_ = 8/group.

Changes in nociceptive sensitivity were measured in rats that have been drinking alcohol for eight weeks in the intermittent access to alcohol in a two-bottle free choice (IA2BC) paradigm. We have previously showed that rats in this paradigm escalate their alcohol consumption and show withdrawal syndrome when alcohol is discontinued^23,24^. The thermal pain sensitivity was significantly higher in rats during 24 h of withdrawal (EtOH-WD) than that of ethanol naive counterparts (CTRL group) (as measured by paw withdrawal latency (PWL) in the Hargreaves test) (Unpaired *t*-test, *t* = 3.9, p = 0.0003, Fig. 1A). In parallel, the basal rate of spontaneous spiking was significantly higher in LHb neurons in brain slices of EtOH-WD rats than that from CTRL rats (Unpaired *t*-test, *t* = 3.789, p = 0.0004) (Fig. 1B–D).

### Chemogenetic inhibition of the LHb alleviates hyperalgesia in ethanol-withdrawn rats

To determine whether the LHb contributes to hyperalgesia in ethanol-withdrawn rats, we infected LHb neurons with an adeno-associated viral vector serotype 5 (AAV5) carrying hM4Di. This hM4Di was under the control of a CaMKIIa promoter, which leads to selective expression in excitatory neurons^25–27^ (Fig. 1E). Bath application of CNO (10 μM) significantly decreased spontaneous firings of LHb neurons in slices infected with hM4Di (Fig. 1F). To test whether pain sensitivity involves the LHb, we compared the PWL from rats infected with hM4Di or a control virus (AAV5-CaMKIIa-eGFP), both in the LHb. Administration of CNO (1 mg/kg, i.p.), but not the vehicle, significantly increased the PWL in hM4Di-infected EtOH-WD rats. CNO did not affect rats infected with the control virus. Thus, LHb inhibition could alleviate hyperalgesia (Two-way ANOVA, for group F_2,29_ = 4.021, P = 0.0288, *post-hoc*: *p < 0.05, **p < 0.01 vs. GFP + CNO; ^#^p < 0.05, ^##^p < 0.01 vs. hM4Di + Veh; N_rat_ = 8–12/group, Fig. 1G).

**Figure 2:**
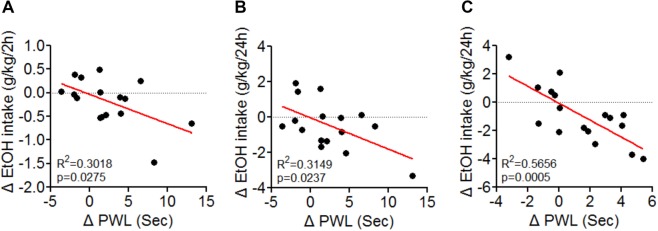
Negative correlation between Paw withdrawal latency (PWL) and Ethanol intake. The changes in PWL induced by LHb manipulation correlated significantly with changes in ethanol intake. Data are presented from rats in GFP/hm4Di expressed group (**A**,**B**) and YFP/KCNQ3 expressed group (**C**) in the LHb. R^2^ and p values are indicated in the plot. (**A**,**B**) N_rat_ = 8/group, (**C**) N_rat_ = 8–9/group.

Also, in hM4Di-infected rats, CNO (1 mg/kg, i.p.) significantly reduced ethanol intake during renewed availability (For 2 h, Unpaired *t*-test, *t* = 2.796, p = 0.0154, Fig. 1H; For 24 h, Unpaired *t*-test, *t* = 2.587, p = 0.0215, Fig. 1I), without altering water intake. There was a significant negative correlation between PWL and the changes in EtOH intake (F_1,14_ = 6.050, p = 0.0275 for 2 h, F_1,14_ = 6.436, p = 0.0237 for 24 h, Fig. 2A,B).

### Chemogenetic activation of LHb neurons induces hyperalgesia in ethanol-naïve rats

**Figure 3:**
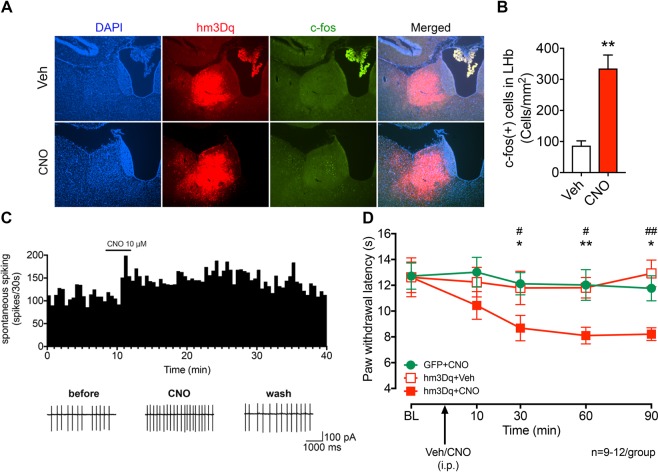
Chemogenetic activation of the LHb induces thermal hyperalgesia in Naïve rats. (**A**,**B**) Representative figures (**A**) and pooled data (**B**) showing that c-fos expression in the LHb is increased after CNO systemic application in hM3Dq overexpressed rats. Unpaired *t*-test, *t* = 4.891, **p < 0.01. N_rat_ = 4–5/group. (**C**) Bath-applied CNO (10 μM) sharply increased the firing rate of LHb neurons infected with AAV-CaMKIIa-hM3Dq-mCherry viruses. (**D**) After systemic CNO injection (1 mg/kg, i.p.), rats infected with hM3Dq showed a decreased paw withdrawal latency than those infected with eGFP. Two-way ANOVA, for group F_2,30_ = 3.583, P = 0.0402, for Time F_4,120_ = 4.534, P = 0.0019, *post-hoc*: *p < 0.05, **p < 0.01 vs GFP + CNO; ^#^p < 0.05, ^##^p < 0.01 vs. hm3Dq+Veh; N_rat_ = 9–12/group.

To ascertain a role of LHb excitatory neurons in nociception, we bilaterally transduced LHb excitatory neurons with CaMKIIa promoter-driven hM3Dq expressing AAV5. CNO (1 mg/kg, i.p.) increased c-fos expression in the hM3Dq-expressing LHb neurons (Unpaired *t*-test, *t* = 4.891, p = 0.0018, Fig. 3A,B). Also, bath application of CNO increased the firing rate of LHb neurons of ethanol naïve rats (Fig. 3C). CNO (1 mg/kg, i.p.) decreased the PWL, indicating that LHb excitatory neurons exert a role in pain-related signaling in both physiological and pathophysiological conditions (Two-way ANOVA, for group F_2,30_ = 3.583, P = 0.0402, for Time F_4,120_ = 4.534, P = 0.0019, *post-hoc*: *p < 0.05, **p < 0.01 vs. GFP + CNO; ^#^p < 0.05, ^##^p < 0.01 vs. hM3Dq + Veh; N_rat_ = 9–12/group, Fig. 3D).

### M-channels in the LHb of ethanol-withdrawn rats are suppressed, and activation of LHb M-channels alleviates hyperalgesia

**Figure 4:**
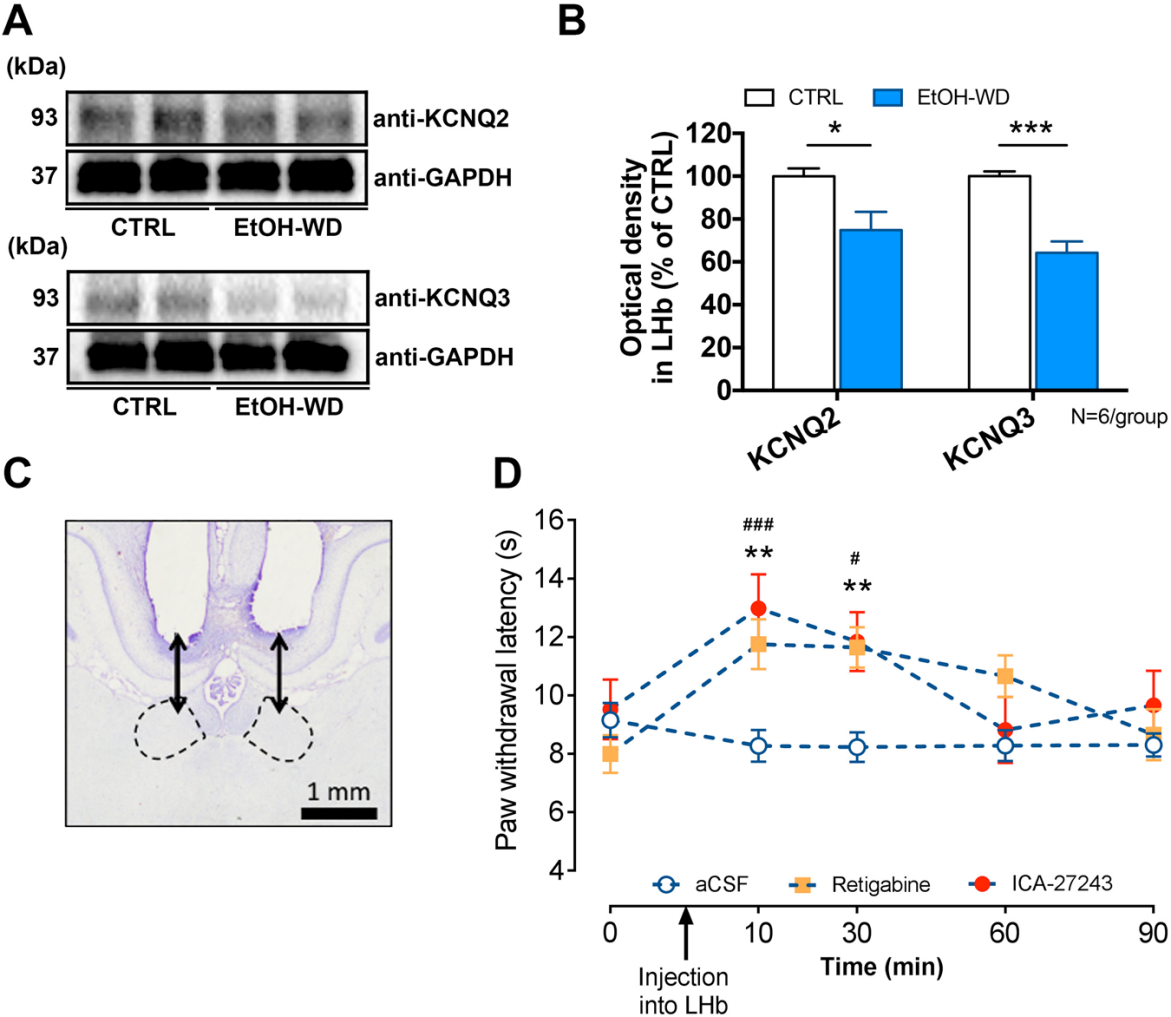
Intra-LHb M-channel activators reduces thermal hyperalgesia in EtOH-WD rats. (**A**–**C**) Representative expression of  KCNQ2 (**A**), KCNQ3 (**B**) and pooled results (**C**) of Western blots show the reduced KCNQ2 and KCNQ3 expression in the LHb from ethanol-withdrawn rats compared to CTRL rats. Note an even greater loss of KCNQ3 expression in the EtOH-WD rats. Unpaired *t*-test, p < 0.05, ***p < 0.001. N_rat_ = 6/group. Full-length blots are presented in Supplementary Fig. 1. (**D**) Cresyl violet-stained brain sections show the accurate guide cannula placements above the LHb. Scale bar = 1.0 mm. (E, F) Intra-LHb retigabine and ICA-27243 increased the paw withdrawal latency (PWL) in EtOH-WD rats (Two-way ANOVA, for Drug F_2,35_ = 0.0017, P = 0.0017, for Time F_4,140_ = 0.6.256, P = 0.0001, *post-hoc*: **p < 0.01 vs. Retigabine, ^#^p < 0.05, ^###^p < 0.001 vs. ICA-27243, N_rat_ = 19(aCSF), 12(Retigabine), 7(ICA-27243).

The above results demonstrate that inhibition of LHb excitatory neurons alleviates hyperalgesia in EtOH-WD rats. To investigate the underlying mechanisms, we examined the m-type potassium channels (M-channels). M-channels are abundantly expressed in the LHb^28^ and are involved in numerous physiological and pathological processes^29^, and may contribute to pain^30^. We previously demonstrated that the expression of KCNQ2/3, the m-channel subunits and the sensitivity to M-channel blocker, XE991, in the LHb of ethanol-withdrawn rats, are reduced^22^. To test whether LHb M-channels are involved in hyperalgesia, we first examined the expression of M-channels in the LHb of rats during 24 h withdrawal. We found that the protein levels of both KCNQ2 and KCNQ3 were significantly decreased (Fig. 4A,B; For KCNQ2, Unpaired *t*-test, *t* = 2.713, p < 0.0218; For KCNQ3, Unpaired *t*-test, *t* = 6.178, p = 0.0001) and the reduction of KCNQ3 was greater (KCNQ2 vs. KCNQ3; Paired *t*-test, *t* = 2.703, p = 0.0426). We then examined the effect of the M-cannel activator, retigabine, an agent that could reduce firing of LHb neurons in ethanol withdrawn-rats^22^. Intra-LHb infusion of retigabine at a dose that decreases ethanol consumption in rats^22,31^, significantly increased the PWL in EtOH-WD rats (Two-way ANOVA, for Drug F_2,35_ = 7.683, P = 0.0017, for Time F_4,140_ = 6.256, P = 0.0001, *post-hoc*: **p < 0.01 vs. aCSF, N_rat_ = 7–19/group, Fig. 4C,D). This increase was attenuated by intra-LHb infusion of ICA-27243 (10 ng in 200 nl/side), a specific KCNQ2/3 activator^32^, (*post-hoc*: ^###^p < 0.001, ^#^p < 0.05 vs. aCSF; p > 0.05, Retigabine vs. ICA-27243), suggesting that the KCNQ2/3 channels in the LHb is the major player in M-channel modulation of nociception.

### Overexpression of KCNQ3 reduces hyperalgesia and ethanol consumption

**Figure 5:**
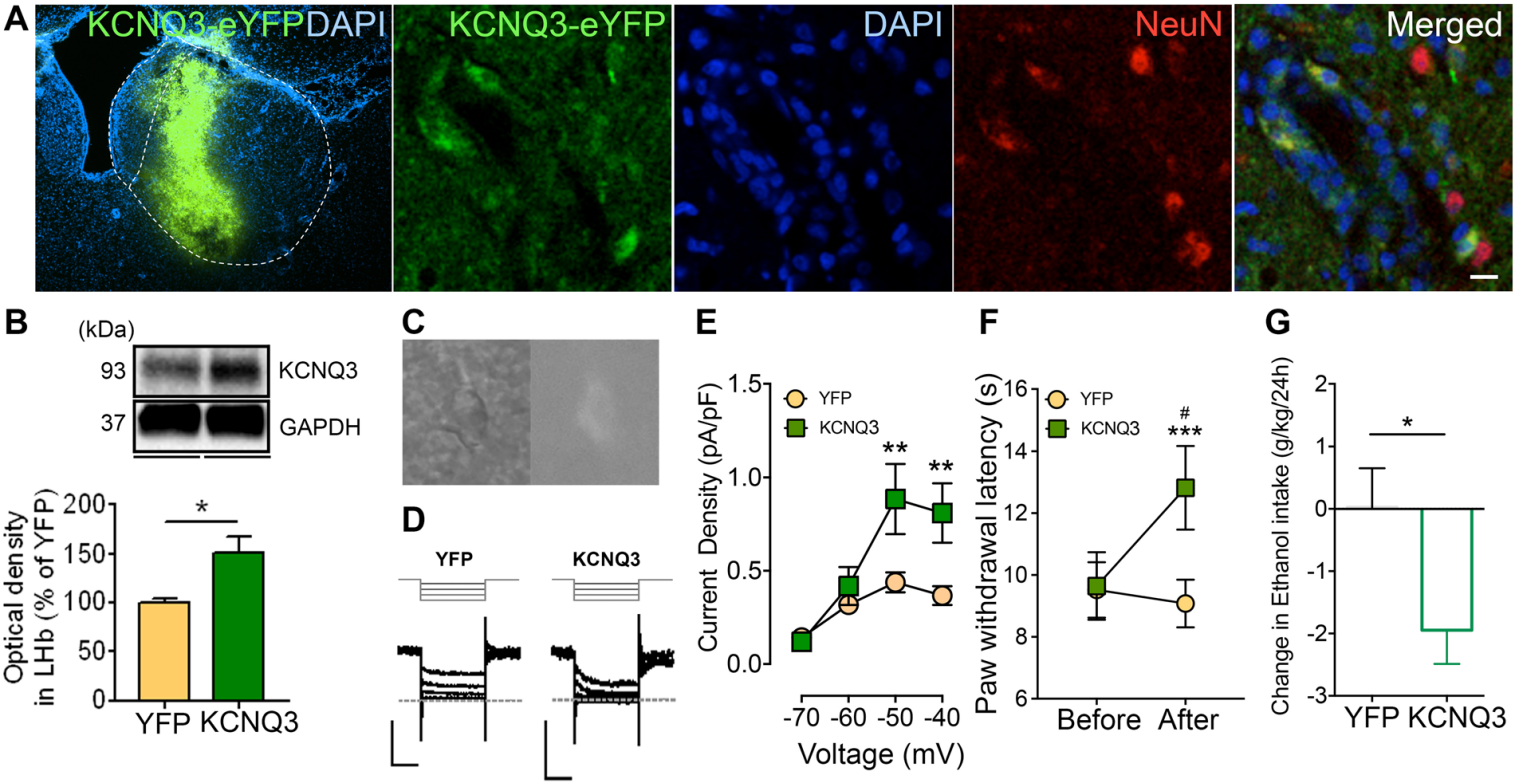
Overexpression of KCNQ3 in the LHb of EtOH-WD rats attenuated thermal hyperalgesia and ethanol consumption. (**A**) KCNQ3-YFP expression after viral vector injection and immunofluorescence of the neuronal marker, NeuN. A Strong signal of KCNQ3-YFP overlaps with NeuN. Scale bar: 10 μm. (**B**) Example and pooled results of Western blots show the increased KCNQ3 expression in the LHb from EtOH-WD rats injected with KCNQ3-eYFP viruses compared to that injected with eYFP control viruses. Unpaired *t*-test, *t* = 3.18, *p < 0.05, N_rat_ = 4/group. Full-length blots are presented in Supplementary Fig. 1. (**C**) CCD camera captured IR (left) and ET-DSRed filtered fluorescence (right) image of the LHb neuron after viral injection for patch-clamp recording. (**D**,**E**) Sample traces (**D**) and pooled data (**E**) showing increased inward current relaxations in neurons of EtOH-WD rats after KCNQ3-eYFP virus injection. Two-way ANOVA, for YFP/KCNQ F_1,17_ = 5.485, P = 0.0316, *post-hoc*: **p < 0.01. N_cell_ = 9–10/group. Scale bar: 200 pA and 200 ms. (**F**) Paw withdrawal latency was significantly increased after KCNQ3 overexpression in the LHb. Two-way ANOVA, for Before/After F_1,16_ = 9.211, P = 0.0079, for Interaction F_1,16_ = 16.06, P = 0.0010, *post-hoc*: ***p < 0.001 vs. Before, ^#^p < 0.05 vs. YFP, N_rat_ = 8–10/group. (**G**) KCNQ3-eYFP-overexpressing rats showed reduced ethanol intake relative to eYFP-overexpressing rats. Unpaired *t*-test, *t* = 2.342, *p < 0.05. N_rat_ = 8–9/group.

To directly test whether rescue of M-channels in the LHb could alleviate hyperalgesia in EtOH-WD rats, we measured the PWL after overexpression of KCNQ3 in the LHb. We used a CRE-dependent FLEX-HSV, which was inactive and required activation by Cre recombinase, to initiate the expression of KCNQ3 and eYFP^33^. We injected this HSV together with an HSV utilizing the HSV IE 4/5 promoter expressing Cre recombinase^34^, which led to a robust expression of KCNQ3 (Unpaired *t*-test, *t* = 3.18, p = 0.0191, N_rat_ = 4/group, Fig. 5A,B). KCNQ3 overexpression in the LHb increased the slow inward relaxation, which reflects the closing of the voltage-dependent M-current (Two-way ANOVA, F_1,17_ = 5.485, P = 0.0316, *post-hoc*: **p < 0.01, N_cell_ = 9–10/group, Fig. 5C–E). Consistent with the effect of pharmacological modulation mentioned above, following KCNQ3 overexpression in the LHb, the PWL was significantly increased (Two-way ANOVA, for Before/After F_1,16_ = 9.211, P = 0.0079, *post-hoc*: ***p < 0.001 vs. Before, ^#^p < 0.05 vs. YFP, N_rat_ = 8–10/group, Fig. 5F). Furthermore, in KCNQ3-overexpressed rats ethanol intake was significantly reduced (Unpaired *t*-test, *t* = 2.342, p = 0.0334, Fig. 5G), and ethanol intake was negatively correlated with the PWL (F_1,15_ = 19.53, p = 0.0005, Fig. 2C).

## Discussion

In this study, we demonstrated that manipulation of LHb neuronal activity altered thermal pain sensation in rats withdrawn from intermittent voluntary alcohol drinking. Specifically, silencing LHb excitatory neurons attenuated thermal hyperalgesia in EtOH-WD rats, whereas chemogenetic activation of these neurons provoked hyperalgesia in ethanol-naïve rats. We also confirmed our published finding of reduced expression of M-channel subunits in the LHb of EtOH-WD rats. We further showed that pharmacological activation or genetic upregulation of M-channels in the LHb alleviated hyperalgesia in EtOH-WD rats. These results suggest that suppression of M-channels in the LHb contributes to the hyperactivity of LHb neurons and hyperalgesia of EtOH-WD rats. Thus, the LHb plays a key role in both pain and alcohol-reward processing.

We also identified molecular adaptations in LHb neurons during ethanol withdrawal. Ion channels are chief modulators of neuronal excitability. Among them, potassium channels act as a break for overflow firing. Consistent with previous findings that expression of M-channel subunits, KCNQ2/3, is reduced in some brain regions of rodents that chronically exposed to ethanol^22,35^, the current study showed that the KCNQ2/3, which are abundantly expressed in the LHb^22,28^, were reduced in EtOH-WD rats. The hyperalgesia observed in EtOH-WD rats was attenuated by intra-LHb infusion of M-channel activators or by overexpression of KCNQ3 in the LHb. Although M-channels have been identified as a modulator of pain transmission in the peripheral neuronal pathway^30,36^, our study shows a role of M-channels in the central nervous system (CNS) in pain sensation.

Human brain imaging and animal studies have shown that the LHb undergoes structural and functional changes with pain and long-lasting hypersensitivity^18,20,37^. Chronic pain is thought to be caused by aberrant neuronal responses along the pain transmission pathway from the dorsal root ganglion to the spinal cord, thalamus, and cortex^38,39^. The sensitization of peripheral nociceptors leads to hyperalgesia. However, the functional and morphological changes in the CNS also significantly contribute to the maintenance of chronic pain and its comorbidities. Thus, the crosstalk between sensory and affective components has been suggested to contribute importantly to the relationship of pain and psychiatric conditions. The LHb has been implicated in promoting behavioral avoidance due to its role in aversion^40,41^.

The major contribution of this study is the identification of LHb excitatory neurons as regulators of thermal hypersensitivity. We took advantage of chemogenetic approaches to selectively regulate LHb excitatory neurons under the control of a CaMKIIa promoter. Compared with traditional excitotoxic lesions and pharmacologic interventions, chemogenetic approaches have the advantage of greater ability to transiently regulate a specific population of neurons^42^. Our observation that silencing LHb excitatory neurons reduced thermal hypersensitivity is consistent with previous pharmacologic intervention studies^9,21^.

Additionally, we showed apparent hyperalgesia in naïve rats in which the LHb was activated chemogenetically, as shown by the reduced PWL in the Hargreaves test. This finding indicates that the LHb has an important role in nociception. Recently, the LHb has been gaining more attention because of its role in psychiatric disorders such as anxiety and depression. Evidence has indicated an association between the LHb and depressive-like behaviors in a mouse model of neuropathic pain^18^ and a rat model of alcohol drinking^43^. Moreover, patients suffering from chronic pain also tend to suffer from depression, and importantly, patients who are diagnosed with depression also commonly suffer from chronic pain. Thus, depression is considered a positive predictor of the development of chronic pain^44^. In this view, LHb may act as a general hub for the connection between pain and depression.

How does the LHb regulate thermal sensitivity? Although the detailed nociceptive circuitry, which allows the LHb to signal to the dorsal horn of the spinal cord to regulate thermal sensitivity, has not yet been established, it may involve several potential signal cascades. Central nociceptive regulation can be achieved through a descending pathway. This pathway starts from the periaqueductal gray (PAG) to the rostral ventromedial medulla (RVM) and reaches to the superficial laminae of the spinal dorsal horn (SDH) (referred to as PAG-RMV-SDH pathway)^45,46^. This pathway comprises an essential neural circuit that exerts powerful modulatory influences on pain^47^. PAG has been shown to mediate thermal hyperalgesia in alcohol-dependent rats^48^. The LHb may regulate the thermal sensitivity^20^ through its projections to the PAG^49^.

Also, morphological, behavioral and electrophysiological evidence suggests the involvement of several neurotransmitters in the analgesia pathway under both physiological and pathological conditions^50–52^. In the central analgesic system descending serotonergic pathways, serotonergic neurons in the raphe nuclei have been considered an important component^53^. Its descending projections, either directly or via the nucleus raphe magnus, modulate the responses triggered by noxious stimulation of the spinal dorsal horn neurons^54^. The LHb has a robust projection to the dorsal raphe (DR) and modulates the activity of DR neurons^14,55,56^. Therefore, withdrawal-induced changes in LHb-raphe circuits may also have an important role in the modulation of thermal responses to noxious stimuli. Further studies are needed to test these possibilities.

Whether pain sensitivity alters alcohol drinking or alcohol drinking alters pain sensitivity through the habenula is still an open question. Alcohol intake by the rats in the IA2BC paradigm was increased after one week in the paradigm (three sessions) but the increase in pain sensation was more apparent after four weeks of alcohol exposure (data not shown), which suggest that, at least in the IA2BC paradigm, repeated alcohol drinking and abstinence seem to alter pain sensitivity. To understand this better, in the future study, we will activate habenula that has been shown to increase pain sensitivity and check whether this will lead to increase alcohol consumption.

We manipulated the activity of LHb neurons in free moving rats by a combination of AAV-driven overexpression of hM3Dq or hM4Di DREADDs in the LHb and systemic administration of CNO. Notably, CNO induced a significant increase in c-fos expression in the LHb in ethanol-naïve rats that expressed hM3Dq in the LHb, suggesting that systemic administration of CNO activates LHb neurons. However, we should interpret these results with caution, since a recent study indicates that clozapine, a metabolite of CNO, mediates the effects of CNO on DREADD receptors in the brain^57^, although CNO is the most well-characterized and advisable ligand for DREADDs until other selective ligands are fully characterized^58^. To avoid possible off-target effects of CNO-driven clozapine via non-DREADDs endogenous receptors, we checked CNO effects in rats without DREADDs expression. Further control validations such as dose-dependent responses and more control groups such as low-dose clozapine treatment should be performed in future studies.

To modulate neuronal activity, we employed the DREADDs strategy “in general”. Although DREADDs GPCRs are genetically modified based on muscarinic receptors, one of which can suppress the M-current via Gq signaling pathway^59^, the DREADDs may also have different cellular signal cascades via various potassium channels to change neuronal activities. Given that M-channels are slowly closing at close-to-resting membrane potentials (RMP) and modulating afterhyperpolarization (AHP)^60–62^ and that repeated alcohol exposure reduces the AHP of the LHb^22^, M-channels of LHb excitatory neurons may play a key role in the adaptation induced by ethanol withdrawal. However, the adaptation may also involve other potassium channels. For example, it has been demonstrated that Kir4.1, one of the inwardly rectifying potassium channels that is predominantly localized in non-neuronal glial cells^63^ and has been known to be reduced in reward-related brain regions after repeated alcohol exposure^35^, modulates the pathological firing patterns of LHb neurons^64^. Therefore, it will be of interest to further investigate the changes in other potassium channels including other KCNQ subunits and their co-factors in the LHb during alcohol withdrawal.

We provided several lines of evidence about the impact of the LHb on hyperalgesia in rats withdrawn from chronic voluntary ethanol drinking. The increased sensitivity to thermal stimuli was concomitant with the increased basal firing rate of LHb neurons and the inhibition of M-channels. Pharmacological activation of LHb M-channels or chemogenetic inhibition of LHb neurons reduced hyperalgesia and ethanol intake. Conversely, chemogenetic activation of LHb neurons increased pain sensitivity in ethanol-naïve rats. Collectively, these findings suggest that M-channels play an important role in the hyperactivity of LHb neurons of ethanol-withdrawn rats, and LHb activity plays a crucial role in hyperalgesia and relapse-like drinking. Manipulating LHb activity through M-channels may be of therapeutic value in the treatment of hyperalgesia and relapse drinking.

## Materials and Methods

### Animals

All studies were conducted on adult male Long Evans rats (n = 157). All procedures and methods were performed by the National Institutes of Health guidelines and regulations with the approval of the Institutional Animal Care and Use Committee of Rutgers, the State University of New Jersey, New Jersey Medical School. The rats were individually housed in ventilated Plexiglas cages in climate-controlled rooms (20–22 °C). The rats had unlimited access to food and water (or as otherwise indicated) and acclimatized to the housing conditions and handling before the start of the experiments. They were kept on a 12 h light/dark cycle.

### Intermittent access to 20% ethanol two-bottle free choice drinking (IA2BC)

Male Long Evans rats (2-month-old, 280–320 g at the start of the experiments) were trained to drink ethanol in the intermittent access in two-bottle free choice (IA2BC) paradigm as previously described^23,24,65^. Briefly, rats were given 24 h concurrent access to one bottle of 20% (v/v) ethanol in tap water and one bottle of plain water, starting at 15:00 on Mondays. After 24 h, the ethanol bottle was replaced by a second water bottle that was available for the next 24 h. This pattern was repeated on Wednesdays and Fridays. On all other days, the animals had unlimited access to two bottles of water. In each ethanol drinking session, the placement of the ethanol bottle was alternated to control for any side preferences. The amount of ethanol or water consumed was determined by weighing the bottles before and after 24 h of access. Ethanol intake was determined by calculating the grams of alcohol consumed per kilogram of body weight.

### Stereotaxic surgery and microinjections

Stereotaxic surgery for pharmacological or genetic modulation of the LHb and its histological verification were performed as described^22,66,67^. A bilateral guide cannulae (FIT 5MM C232G-1.5W-1MM PROJ, 22 gauge; Plastics One, Roanoke, VA, USA) was inserted 1 mm dorsal to the LHb (AP: −3.83 mm, ML: ± 0.63 mm, DV: −4.45 mm). The injecting position was calculated based on Paxinos and Watson atlas^68^. Rats resumed alcohol drinking seven days after surgery. Vehicle or retigabine (10 ng in 0.01% dimethyl sulfoxide (DMSO) v/v aCSF), at a dose shown to affect ethanol intake^22,31^, was injected into the LHb (200 nl/side at a rate of 200 nl per min).

### Intra-LHb virus injection and clozapine-n-oxide (CNO) treatment

We expressed the engineered human muscarinic receptor (DREADDs, designer receptors exclusively activated by designer drugs) to inhibit or excite neuronal activities. Either of AAV5-CaMKIIa-hm4Di-mCherry, AAV5-CaMKIIa-hm3Dq-mCherry, or control AAV5-CaMKIIa-eGFP (titers of 10^12^−10^13^ vg/ml, UNC Vector Core, Chapel Hill, NC) were injected bilaterally into the LHb (AP: −3.83 mm, ML: ± 0.63 mm, DV: −5.45 mm) of rats. A volume of 420 nl per side was delivered at a rate of 70 nl min^−1^. CNO (1 mg/kg, dissolved in 0.5% DMSO v/v saline, intraperitoneal injection, i.p.) was given 10 minutes before the behavioral tests, 24 h after the last ethanol session. After five weeks of IA2BC paradigm, AAV virus injections were performed. One week after recovery, rats were returned to the IA2BC paradigm and exposed to the paradigm three weeks further. After four to five weeks of AAV virus injection, the behavioral tests were performed.

For overexpression of KCNQ3 in the LHb, we infused into the LHb Cre dependent HSV-LS1-KCNQ3-eYFP virus (gift of Ming-Hu Han in Mount Sinai^33^) and HSV-IE4/5-CRE virus (from Dr. Rachael Neve, Viral Gene Transfer Core, MIT). HSV-LS1-KCNQ3-eYFP or HSV-LS1-eYFP were mixed in a 2:1 ratio with HSV-IE4/5-CRE before injection. The HSV virus were injected into rats that were in the IA2BC paradigm for seven weeks. Three days after injection, rats were returned to the same IA2BC paradigm for an additional one week before the tests were conducted.

### Pain threshold measurement (Hargreaves test)

Hargrave’s test has been used to assess hyperalgesia in alcohol-depedent rats^48^. Paw withdrawal latencies (PWL) were measured with an Analgesia Meter (Model 336; IITC Life Science Instruments, Woodland Hills, CA) as described^8,9,69^. Briefly, each rat was placed in a plexiglass chamber on a glass plate located above a light box. The temperature of glass was set to 25 °C. After an acclimation time to the environment, these rats were subjected to radiant heat, which was applied by aiming a light beam to the middle of the plantar surface of the hind paw. When the rat lifted its paw in response to the heat, the light beam was turned off, and the PWL was recorded. A cutoff time of 20 seconds was used to prevent paw tissue damage. We measured the PWL once a week, at both 2 h and 24 h after the last ethanol drinking session. The two measurements were conducted consecutively, the same PWL changes were used for Fig. 2A,B to determine the correlation between alcohol drinking and pain sensitivity.

### Brain slice preparation and electrophysiology

Coronal epithalamic slices (300 μm) were cut in ice-cold artificial cerebral fluid (aCSF) containing (in mM): 126 NaCl, 2.5 KCl, 1.25 NaH2PO4, 1 MgCl2, 2 CaCl2, 25 NaHCO3, 1 l-ascorbate and 11 glucose, and saturated with 95% O_2_/5% CO_2_ (carbogen). Slices were then incubated for >1 h at 24–25 °C in carbogenated aCSF. The LHb region was identified as described^70,71^. The spontaneous firing and M-currents were recorded with the loose-patch cell-attached technique and voltage clamping, respectively, in warm (~33 °C) aCSF perfused at 2–2.5 ml/min. Patch pipettes (6–8 MΩ) were filled with the solution containing (in mM) 130 K-methanesulfonate, 10 KCl, 4 NaCl, 10 HEPES, 0.5 EGTA, 2 MgATP and 0.2 Na2GTP. M-currents were recorded in the presence of 1 µM tetrodotoxin, at a holding potential of −30 mV to activate the current and deactivated by 500-ms repolarizing steps. Membrane potentials were corrected for liquid junction potential (+8.8 mV).

### Western blotting and antibodies

Tissue containing bilateral LHb was punched out from coronal slices (400-μm thick) and homogenized in ice-cold RIPA buffer (Sigma Aldrich, St. Louis, MO) containing a protease inhibitor cocktail (Sigma Aldrich). Equal amounts of protein extracts were denatured and subjected to SDS-polyacrylamide gel electrophoresis. Protein-separated PVDF membranes (Bio-Rad, Philadelphia, PA) were blocked with 5% non-fat milk in TBST ((in mM) 24 Tris, pH 7.4, 137 NaCl, 2.7 KCl, and 0.05% Tween 20) for 1 h at room temperature. Then these membranes were incubated with anti- KCNQ2 antibody (1:800, Alomone, Jerusalem, Israel), anti-KCNQ3 antibody (1:800, Alomone), or anti-GAPDH antibody (1:3000, Sigma Aldrich, St. Louis, MO) overnight at 4 °C. After washing with TBST, the membranes were incubated for 1 h with Goat anti-rabbit IgG-HRP conjugated antibody (1:2500, Jackson Immuno-Research, West Grove, PA) at room temperature. The proteins were visualized by ECL solution (Perkin Elmer, Waltham, MA) using the ChemiDoc XRS system with Image Lab software (Bio-Rad).

### Immunofluorescence

Anesthetized rats were transcardially perfused with saline followed by 4% paraformaldehyde (PFA). After post-fixation (overnight, at 4 °C) in 4% PFA, cryoprotected tissue was sliced (30-μm thick) using a microtome (Microm HM550, Walldorf, German). Primary antibodies against NeuN (Mouse, 1:2000, EMD Millipore), Goat anti-mouse IgG Fluorescein (Vector), Goat anti-mouse IgG Cy3 (Jackson) at 1:400 dilutions were used. Immunopositive cells were visualized with a Nikon Eclipse A1 confocal microscope (Nikon, Melville, NY).

### Drugs

We purchased common chemicals from Sigma Aldrich (St. Louis, MO, USA) except retigabine from Alomone (Jerusalem, Israel) and ethanol from Pharmco Products Inc. (Brookfield, CT). Clozapine-N-oxide (CNO) was provided from NIH by the NIDA Drug supply program (NIH, Bethesda, MD).

### Data analysis and statistics

We decided the number of N in the experiments according to the previous our studies and literatures. We compared the mean frequency of spontaneous firing over the last 3-min of 5-min periods of recording and calculated the drug-induced changes after normalizing the data to the preceding 3-min of baseline firing. To determine a correlation between alcohol drinking and pain sensitivity in rats, we calculated the changes in paw withdrawal latency (delta PWL) by subtraction of the response after CNO to the response after SAL at the time point of 1 hour after drug injections (i.p.). Prism (GraphPad, La Jolla, CA) was used for statistical analyses. All compiled data were shown as mean ± SEM and the statistical significance was assessed using paired or unpaired *t-*tests, and one- or two-way ANOVA with *post hoc* multiple-comparisons, when appropriate. Linear regression was used to determine the relationship between change in paw withdrawal latency and change in ethanol intake. The values were considered significant when P < 0.05.

## Supplementary information

Suplementary info

## Data Availability

The authors declare that all data supporting findings of this study are available within the paper and its supplementary information files are available from the corresponding author upon request.
