# Supplementary material for: Downregulation of M-channels in lateral habenula mediates hyperalgesia during alcohol withdrawal in rats
Source: Sci Rep. 2019 Feb 25;9:2714. doi: 10.1038/s41598-018-38393-7 (PMC6389965; doi:10.1038/s41598-018-38393-7)

Downregulation of M-channels in lateral habenula mediates hyperalgesia during alcohol withdrawal in rats

Seungwoo Kang[1,2,3], Jing Li[1,2], Wanhong Zuo[1,2], Pei Chen[1,2], Danielle Gregor[1,2], Rao Fu[1,2], Xiao Han[1,2], Alex Bekker[1,2], Jiang-Hong Ye[1,2,*]

Supplementary Figure 1 Representative full images of immunoblotting. Boxed areas were cropped for designated figures. Dotted lines denote separations of membranes for dual labeling.

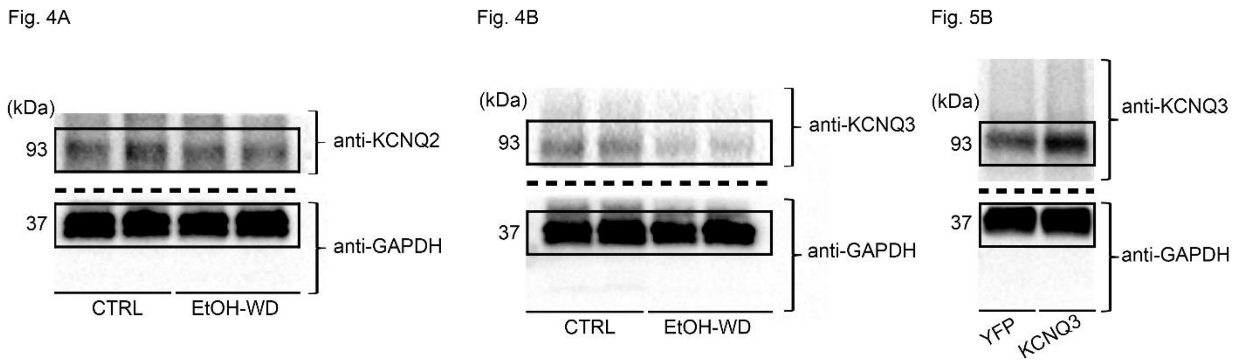

Supplement: Supplementary file 1 — Suplementary info [file 41598_2018_38393_MOESM1_ESM.pdf]
